# Supplementary material for: Causal Relationship between Aging and Anorexia Nervosa: A White-Matter-Microstructure-Mediated Mendelian Randomization Analysis
Source: Biomedicines. 2024 Aug 16;12(8):1874. doi: 10.3390/biomedicines12081874 (PMC11351342; doi:10.3390/biomedicines12081874)
Supplement: Supplementary file 1 [file biomedicines-12-01874-s001.zip › Supplementary Figure/Figure S4/Singe SNP plot of MR analysis for TL to ubm-b-1453.pdf]

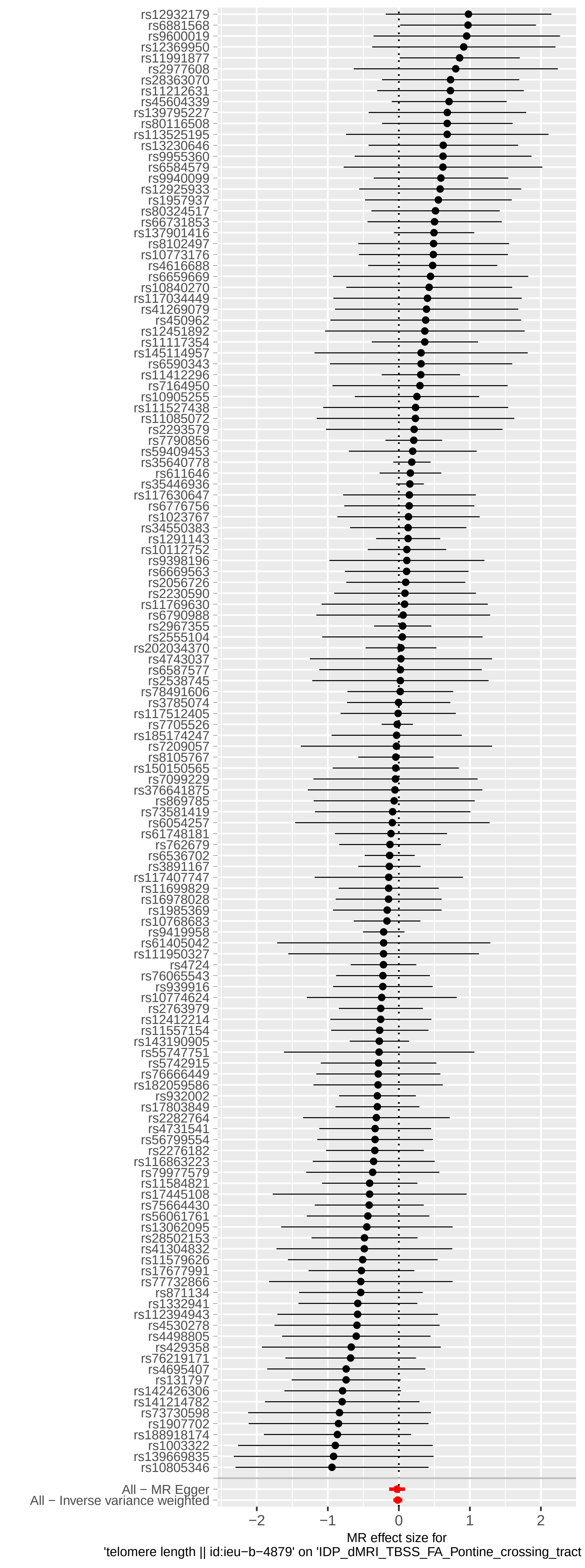

rs12932179
rs6881568
rs9600019
rs12369950
rs11991877
rs2977608
rs28363070
rs11212631
rs45604339
rs139795227
rs80116508
rs113525195
rs13230646
rs9955360
rs6584579
rs9940099
rs12925933
rs1957937
rs80324517
rs66731853
rs137901416
rs8102497
rs10773176
rs4616688
rs6659669
rs10840270
rs117034449
rs41269079
rs450962
rs12451892
rs11117354
rs145114957
rs6590343
rs11412296
rs7164950
rs10905255
rs111527438
rs11085072
rs2293579
rs7790856
rs59409453
rs35640778
rs611646
rs35446936
rs117630647
rs6776756
rs1023767
rs34550383
rs1291143
rs10112752
rs9398196
rs6669563
rs2056726
rs2230590
rs11769630
rs6790988
rs2967355
rs2555104
rs202034370
rs4743037
rs6587577
rs2538745
rs78491606
rs3785074
rs117512405
rs7705526
rs185174247
rs7209057
rs8105767
rs150150565
rs7099229
rs376641875
rs869785
rs73581419
rs6054257
rs61748181
rs762679
rs6536702
rs3891167
rs117407747
rs11699829
rs16978028
rs1985369
rs10768683
rs9419958
rs61405042
rs111950327
rs4724
rs76065543
rs939916
rs10774624
rs2763979
rs12412214
rs11557154
rs143190905
rs55747751
rs5742915
rs76666449
rs182059586
rs932002
rs17803849
rs2282764
rs4731541
rs56799554
rs2276182
rs116863223
rs79977579
rs11584821
rs17445108
rs75664430
rs56061761
rs13062095
rs28502153
rs41304832
rs11579626
rs17677991
rs77732866
rs871134
rs1332941
rs112394943
rs4530278
rs4498805
rs429358
rs76219171
rs4695407
rs131797
rs142426306
rs141214782
rs73730598
rs1907702
rs188918174
rs1003322
rs139669835
rs10805346
All − MR Egger
All − Inverse variance weighted
−2
−1
0
1
2
MR effect size for
'telomere length || id:ieu−b−4879' on 'IDP_dMRI_TBSS_FA_Pontine_crossing_tract
